# Effects of 8-Week Complex Balance Training in Young Alpine Skiers: A Pilot Study

**DOI:** 10.1155/2018/6804534

**Published:** 2018-11-21

**Authors:** Kajetan J. Słomka, Michał Pawłowski, Justyna Michalska, Anna Kamieniarz, Anna Brachman, Grzegorz Juras

**Affiliations:** Department of Human Motor Behavior, The Jerzy Kukuczka Academy of Physical Education in Katowice, 72A Mikolowska Street, 40-065 Katowice, Poland

## Abstract

**Objectives:**

The purpose of this study was to determine the effects of an 8-week complex balance training program on dynamic balance in skiers according to the new balance training protocol.

**Design:**

Intervention study, comprising 8 weeks of core stability, plyometric, balance, and stretching exercises.

**Participants:**

Ten young skiers volunteered to take part in this study (average age, height, and body mass were 16,44 +/- 1,07 years, 172,76+/-8,84 cm, and 67,4 11,44 kg, respectively (mean +/- SD)).

**Main Outcome Measures:**

Subjects' dynamic balance performance was assessed and retested after training completion with the use of dynamic balance measurement within the Optojump Next System.

**Results:**

The results of 8-week complex balance training showed significant improvements for jump height (H) and flight time (FT) for the left leg and jumping area in both legs.

**Conclusion:**

The complex balance training program improved parameters of dynamic stability in young skiers and led to decreased asymmetry between lower extremities.

## 1. Introduction

Regardless of the discipline, sports training proved to have a positive influence on body balance. The high level of balance ability allows achieving many benefits including performance improvement, injury prevention [1–3], and faster rehabilitation [4]. These potential profits make a specific balance training essential for every sport discipline. Recent years show a growing number of implemented balance training protocols in different sports disciplines such as team sports [5–11], downhill skiing [12, 13], figure skating [14], capoeira [15], tennis [16], running [17], field hockey [18], and gymnastics [19]. Due to this diversity and different specificity of sports the gold standards of balance training and exercises, common for most situations, seem not to exist [2]. The protocols mentioned above were not always defined and described. The terms used in the descriptions were sometimes misleading and confusing. The following terms are most commonly used: balance and core stability exercises [20–23], neuromuscular and proprioceptive training with mixed strength, balance and plyometric exercises [4, 6, 13, 23], and sensorimotor training [24, 25]. The differences in training volume are also substantial. Some authors used full training units [22, 26, 27]; others used only a warm-up [19, 28, 29]. One can also find in the literature specific suggestions for balance training application [3, 30]. These review studies define training modalities such as exercises time and frequency, number of sessions, duration of a single training session, number of exercises and sets per training session, and effective training dose.

Skiing is considered an extreme sport of high-risk injury incidence, where the most prevalent are the knee injuries [31]. The contemporary style of skiing emphasizes, above all, the speed and movement technique of the skier [32]. Apart from energy expenditure requirements, skiing demands high level of motor abilities (e.g., speed, balance) [33, 34]. The neuromuscular system must be adequately trained for balance to enable efficient movement down the ski slope and to prevent falls [35]. Falls during ski training and competition can lead to the occurrence of multiple injuries of different etiology [32]. Just like any other sports with high speed and strength demands skiing motivates high level of motor control over the narrow base of support (skis). Specifically, the postural control should be emphasized during the training which in consequence leads to better balance during performance. One can speculate that this will decrease falls occurrence and injuries. Since skiing is very dynamic in nature essentially the dynamic balance control is needed. To address this issue one should incorporate dynamic exercises, which are focused on core stability and plyometrics, into the training [36]. This is important both in young and adult skiers who spend most of their training time on the ski slope working on the skiing technique. The area of motor preparation in sports is in constant development and takes advantage from the current achievement in physiology and biomechanics. Unfortunately, the specificity of alpine skiing forces athletes to devote much time of the training to the on-snow training and less time remains for an effective basic motor preparation [37]. Incorporation of specific balance training in their routine could be another factor improving overall performance [38]. Therefore, the aim of this study was to examine the influence of specific balance training on dynamic balance in elite polish young skiers. We hypothesize that the proposed balance training will elicit changes in skiers' dynamic balance performance.

## 2. Materials and Methods

### 2.1. Experimental Approach to the Problem

The study was designed to evaluate the effects of a complex balance training program on dynamic balance in skiers. The program comprised exercises focused on core stability, plyometrics, balance with devices, and stretching. The training was executed twice a week, along with the regular practice. All the subjects were tested with the use of Drift protocol within the Optojump Next System (Microgate, Italy), which is designed to evaluate dynamic balance. The measurements were taken before and after the 8-week training program.

### 2.2. Subjects

Ten top young polish skiers with FIS points under 100 and competing on the national and international level volunteered to take part in this study. The skiers were under a special athletic program in the school being the center of alpine skiing preparation for youth.

Their average age, height, and body mass were 16,44 +/- 1,07 years, 172,76+/-8,84 cm, and 67,4 11,44 kg, respectively (mean +/- SD). Athletes, parents, and coaches were informed about the aims of the study, and written informed consent was acquired from the subjects prior to their participation in the investigation. The study was approved by the Institutional Ethic Board.

### 2.3. Testing Procedures

Subjects' balance performance was assessed prior to 8-week balance training program and retested after its completion with the use of Drift protocol included within testing procedures in the Optojump Next System, which has been proved to be valid and reliable [39]. The test was designed to evaluate athletes' jumping performance, asymmetries, and dynamic balance. The following variables were analyzed: jump height (H) [cm], power (Power) [W/Kg], ground contact time during jumps (CT) [s], jump flight time (FT) [s], average displacement of the jumping point during jumps (drift) in the mediolateral direction (DR-ML) [cm], average displacement of the jumping point during jumps (drift) in the anteroposterior direction (DR-AP) [cm], standard deviation of the average drift in ML (Sd-DR-ML) [cm], standard deviation of the average drift in AP (Sd-DR-AP) [cm], and the area occupied during jumping (Area) [cm2]. All parameters were established for both legs separately, denominated by R (right) and L (left) in the variable names. Additionally the differences between the right and left leg in performance were calculated, being the measure of functional asymmetry. Prior to testing, subjects performed a warm-up that consisted of running and dynamic stretching exercises. The Drift test protocol consists of five, consecutive, unilateral, and vertical jumps for the right and left leg. The series of jump were repeated two times (foot parallel and orthogonal to the Optojump bars) in order to achieve the two dimensional picture of the dynamic stability of the jumping performance. To standardize the test execution each subject had to elevate their foot at the knee height of the contralateral leg and keep it at this level during the jumps. Subject's hands were in the akimbo position to eliminate the influence the technique on the jumping performance. The instruction for the subjects was to jump 5 times as fast and as high as they can. The optimal score on Drift test is performed in the smallest area, the highest height of jumps, and the shortest contact time with the ground. It requires a high level of coordination (dynamic balance, core stability) and strength from the subjects.

### 2.4. Training Protocol

The training lasted 8 weeks (twice a week on non-consecutive days) and comprised core stability, plyometric, balance, and stretching exercises. The training was supervised at the beginning by trained physiotherapists and then continued by the instructed coach. Appropriate instructions were given to the athletes throughout the training program. A standardized warm-up routine comprising running and dynamic stretching exercises was used before the training which did not differ from their standard warm-up routine (Table 1). The outline of the training program is presented in Table 2.

### 2.5. Statistical Analysis

The basic descriptive statistics were calculated. The Shapiro-Wilk test was used to verify the normality of the distribution of data. The differences between the analyzed variables pre- and posttraining were estimated with the use of Student's t-test for dependent variables or Wilcoxon signed-rank test where applicable. All calculations were done with the use of the Statistica software package (ver. 12). The level of significance was p<0,05.

## 3. Results

**Figure 1:**
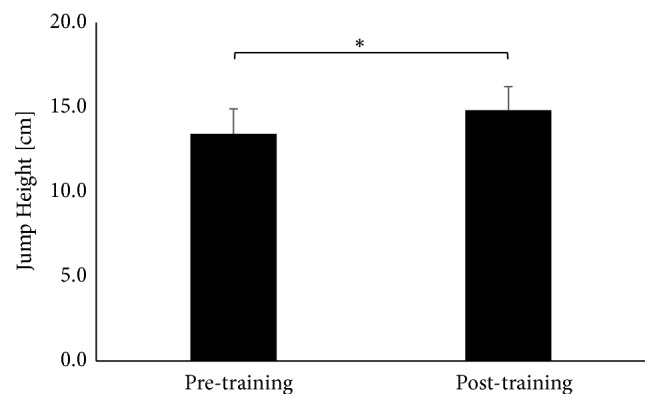
The average jump height for the left leg.

The effects of 8-week complex balance training showed significant differences between pre- to posttests for the dependent variables and were further described in detail. The results of t-test for dependent variables presented a statistically significant improvement in jump height for left leg after the training (t(9)=-3.17, p = 0,011) (Figure 1).

**Figure 2:**
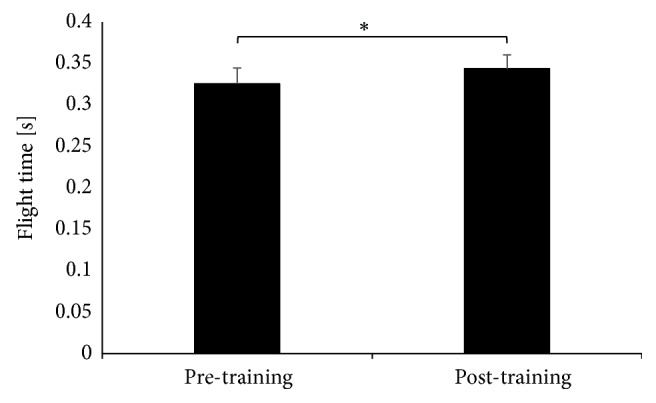
The average flight time for the left leg.

No significant effects in jump height of the right leg were registered (t(9) =-1.61, p=0.141). According to the Wilcoxon signed-rank test results the same was observed for average jump height for both legs (Z=0,05, p< 0,96). We have observed a significant effect in flight time for the left leg post-exercise program (t(9)= -2,95 p =0,016) (Figure 2).

Non-significant changes in flight time for the right leg post-exercise program were observed (t(9)=-1.66, p=0.131) likewise in flight time for both legs (Z=0.25, p< 0.80). The differences after the training in power of the left (t(9)= -1.57, p=0.152); right (t(9)= -0.27, p=0.794); and both legs (Z=0.36, p<0.72) were also not statistically significant. Moreover, we did not observe a significant effect in the contact time for the left leg (Z= 1.58, p<0.11), right leg (t(9)=-1.40 p=0.196); and both legs (Z=0.56, p< 0.58) after the applied training.

**Figure 3:**
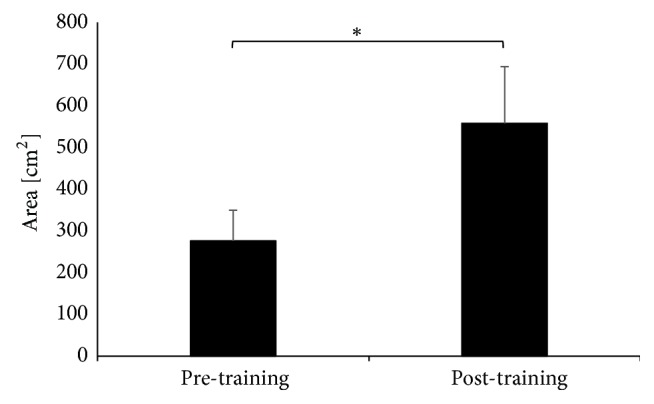
The Area during jumping on left extremity.

**Figure 4:**
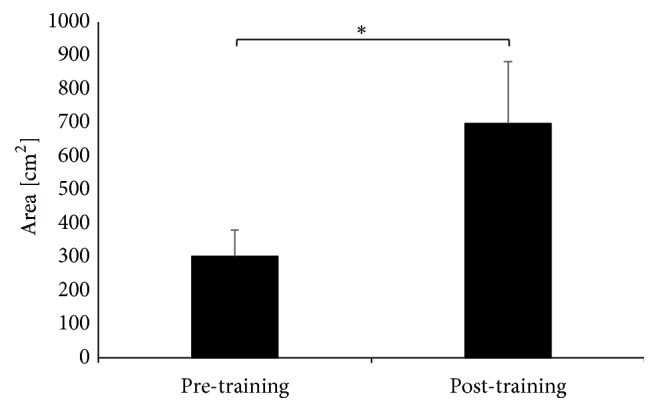
The Area during jumping on right extremity.

The balance training program induced significant changes in the Area variable after the training for the left leg (t(9)=-2.29 p=0.047) (Figure 3) and the right leg (Z=2.09, p<0.04) (Figure 4) during unilateral jumps, but not for both legs jumps (Z=0.87 p<0.39).

We did not find significant differences in average drift in the mediolateral direction after the training for left (t(9)=1.66, p=0,131); right (t(9)=1.64 p=0.136); and for both legs (Z=0.05, p<0.96). Similarly, in the anteroposterior direction, the training effect occurred not to be significant for the left (t(9)=0.38 p=0.713); right (t(9)=2.18 p=0.06); and both legs (Z=1.38, p<0.17), respectively.

The same was observed for the standard deviation of the average drift in ML and in AP directions. The Wilcoxon signed rank test did not show significant differences in ML direction for the left (Z=0.87, p<0.39) and right leg (t(9)=-1.66, p=0.132). In AP direction after the training the results were the following for left leg (t(9)=-1,35 p = 0,210) and right leg (t(9)=-1,86 p = 0,096).

**Figure 5:**
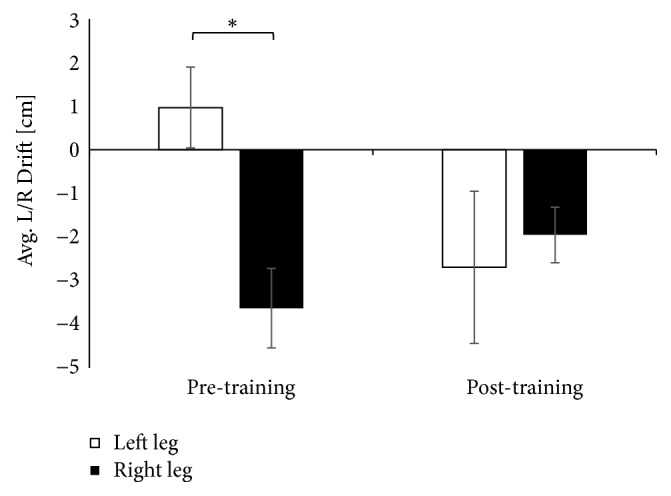
The average drift in the mediolateral direction before and after performing balance training.

Finally the data indicated a decrease in the asymmetry between the lower extremities in the Sd-DR-ML variable after the training, which was present and statistically significant before the training (Figure 5), (for average drift in mediolateral direction U=16, Z=2.53, and p=0.011).

No significant differences after the training program were observed in other variables between left and right leg, which were: jump height (U=43, Z=-0.49, and p=0.623), power (U= 48, Z=0.11 and p=0.909), flight time (U= 43, Z=-0,49, and p=0.623), contact time (U=30, Z=-1.47, and p=0.140), and average drift in anterioposterior direction (U=43, Z=-0.49, and p=0.623), standard deviation of average drift in ML direction (U=44, Z=-0.42, and p=0.678) and AP directions (U=48, Z=-0.11, and p=0.91) and the Area (U=45, Z=-0.34, and p=0.734).

## 4. Discussion

We aimed to examine the influence of a specific balance training on dynamic balance and lower extremities power and strength performance in young alpine skiers. Our hypothesis was that the proposed balance training will elicit significant changes in skiers' dynamic performance and balance. In general our main findings were the significant improvements in the jump height, flight time, and area for the left leg and area for right leg induced by the balance training. Positive changes in jump height and flight time are very closely related. What is interesting is the increase in the occupied area during jumps in both legs. This means that the increase in strength capabilities did not involve improvement in the control processes; the higher the jump the harder to control the behavior of the body. Therefore, we advocate for the importance of the core stability training along with the plyometrics which may lead to improvement in the dynamic control of performance. This aspect can be potentially improved in the examined group.

We have also observed a significant decrease in an average drift in the mediolateral direction after the proposed balance training. The new approach to the balance training proposed by Brachman et al. [30] was verified and its effectiveness for improvement in dynamic stability proved to be positive in the examined group. Our findings emphasize the significant impact of balance training on sport performance (its improvement).

Several studies are in accordance with our results, showing that the introduction of a balance training component to the practice schedule allow for improvement in a wide range of movements, starting from the simple movement such as a vertical jump [40], through the complex ones executed during downhill slalom skiing [13]. Other research presented in the review study by Markovic [41] shows significant improvement in jump height after plyometric training. Similarly, like in our study, there are researches where improvement of balance leads to a decrease in functional asymmetry in performance [40]. Not all balance training programs (e.g., resistance and vibration training) reported in the literature lead to the balance improvement [12]. Comparison of balance and resistance training was done in other studies and the results showed that the resistance training was more beneficial to jump height than the balance training [42]. In our research, we used core stability exercises as a kind of resistance training leading to improvement in sport performance. The effect of training protocol, which we used, should be compared with effects of specific resistance training protocols in the future studies. We are also aware that few days of ski training can induce positive changes in some parameters of balance [43].

Our results seem to support the utility of balance exercises incorporated into the training schedule in the decrease of the lower extremity performance asymmetry being the difference in mediolateral drift between the left and right leg. This constitutes one of the injury prevention goals. We consider this as one of the most significant effect of the conducted 8-week balance training. This was also present in other studies [2–4, 44]. These findings confirm the usefulness of the balance training protocol used in these studies. It is necessary to examine other athletes from different disciplines with different competition level to create the gold standard of balance training procedures. In conclusion, we believe that complex balance training might lead to increase in the dynamic balance and overall performance. This is an important part of sports conditioning that contribute to improved specific performance in skiers.

### 4.1. Study Limitations

Although the study has reached it aims there were some unavoidable limitations. First, the study was conducted on a small number of selected population, which limits our ability to draw more meaningful conclusions. On the other hand it allows for improved design in the future based on sample size and power of the test statistics. Another limitation is the results concerning the performance only of the lower part of the body. It would be very useful to use measurements of the upper body kinematics to better estimate dynamic balance performance. The low number of subjects and the lack of a control group are detrimental to the final results which one can only speculate to be based on the conducted balance training, therefore one should treat this study as a promising pilot. Another limitation of this paper is a difficulty to create an aimed functional training program for a specific group such as skiers. Skiers spend most of the season time on outdoor training in the mountains, so close cooperation with the coaches is essential. We did not control the effects of applied training one week and four weeks after the end of the training program, which would be essential to estimate the retention of these effects. Another limitation is to adequately incorporate this training into the training macrocycle in order to boost the potential effects. The reliability of the standalone Drift test was not established yet; however, there were several studies reporting reliability and validity of the Optojump Next system [39]. We are aware that this is not a trivial question and the high reliability of the measurement system may not convey to the separate tests. This is still to be done in the future.

### 4.2. Practical Applications

The complex balance training program improved some parameters of dynamic stability in young skiers. Moreover, it leads to decreased asymmetry between lower extremities. Balance training protocol of this study should be an essential tool for all sports trainers and coaches.

## Figures and Tables

**Table 1:** Warm-up protocol.

**Exercise**	**Rep**	**t [s]**	**Rest [s]**
Active Warm-up Game	1	180	30
Knee to Chest Walk	10	30	-
Inchworm	5	60	20
Rotational Walking Lunge	5	60	20
Spiderman Crawl	5	60	20
Tuck jump with knees up	5	20	20
Tuck jump with a heel kick	5	20	20
Single leg-hops	10	60	-

**Rep** = *repetitions*; ***t***= *time*; **Rest**= *rest between exercises.*

**(a):** 

Exercises/ Phase	**Phase 1**				**Phase 2**				**Phase 3**			
	**Weeks 1-2**				**Weeks 3-5**				**Weeks 5-8**			
**Core Stability Training**	SxR	t [s]	RS [s]	RR [s]	SxR	t [s]	RS [s]	RR [s]	SxR	t [s]	RS [s]	RR [s]

Front plank	1x2	60	-	20								

Quadruped exercise	1x6	10	-	-								

Clamshell	2x10	5	-	-					2x 5R 2x 5L	10	-	-

Back bridge	1x2	60	-	10								

Back extension	3x5	5	10	-								

Front plank raising right, left arms, right left legs separately					2x4	30	-	-				

Quadruped exercise raising left leg, bend knee (90*∗*) and push up					1x15L 1x15R	-	-	-	1x10L 1x10R	-	-	-

Side bridge					2x10L 2x10R	5	-	-				

Back bridge raising left or right leg					1xR 1xL	60	-	10				

Prone arm and leg lift					3xR 3xL	5	10	-	3x8R 3x8L	5	10	-

Front plank raising right arm and left leg or left arm and right leg									2x4	30	-	-

Back bridge raising leg bend knee and hip to 90*∗* and push up									1 x R 1 x L	30	-	10

**Stretching**	SxR			t [s]	SxR		t [s]		SxR	t [s]		

Latissimus dorsi	2x2			15	2x2		15		2x2	15		

Pectorals/biceps	2x2			10	2x2		10		2x2	10		

Posterior deltoids	2x2			10	2x2		10		2x2	10		

Hip flexors	2x2			10	2x2		10		2x2	10		

Calf stretch	2x2			10	2x2		10		2x2	10		

Quadriceps	2x2			10	2x2		10		2x2	10		

Hamstring	2x2			10	2x2		10		2x2	10		

Iliotibial band/lower back	2x2			10	2x2		10		2x2	10		

**SxR** = *series x repetitions*; **t**= *time*; **RS**= *rest between series*; **RR**= *rest between repetitions*; **R**= *right limb*; **L**= *left limb.*

**(b):** 

Exercises/ Phase	**Phase 1**			**Phase 2**			**Phase 3**		
	**Weeks 1-2**			**Weeks 3-5**			**Weeks 5-8**		
**Plyometric training**	Rep	t [s]	RE	Rep	t [s]	RE	Rep	t [s]	RE

Athletic position	5	5							

Wall jumps	-	15		-	15				

Squat jumps	-	10		-	15				

Tuck jump (thighs parallel)	-	10							

Line jump (side to side)	-	10							

Line jump (lateral max vertical)	-	10							

Lunge jump	-	10							

180 jumps	-	15		-	15				

Broad jump (vertical)	8	-							

Bounding in place	-	20							

Forward jumps over barriers	6	-							

Forward jumps with middle box	6	-							

Box drop jump (max vertical)	10	-							

Tuck jump (with abdominal crunch)				-	15				

Tuck jump (with butt kick)				-	15				

Barrier jumps (front to back)				-	15				

Barrier jumps (side to side)				-	15				

Hop,hop,hop - athletic position				6R; 6L	-				

Broad jump, jump, vertical jump				8	-		8	-	

Bounding for distance				4	-		4	-	

Side jumps over barriers				6R; 6L	-				

Side jumps over bench				6	-				

Drop jump (lateral box max vertical)				8	-				

Squat - tuck jumps							-	15	

Barrier hops flat (front to back)							14R; 14L	-	

Barrier hops flat (side to side)							14R; 14L	-	

Broad jump, jump, vertical jump + step							8	-	

3 barrier hop- reaction (3way)							3R;3L	-	

Forward-backward hops over barriers + step							8R;8L	-	

Box drop 180 box drop max vertical + step							15	-	

Ski jumps							-	15	

**Rep** = *series x repetitions*; **t** = *time*; **RS** = *rest between series*; **RR** = *rest between repetitions*; **R** = *right limb*; **L** = *left limb*; **R** = *repetitions*; **RE** = *rest before next task*.

**(c):** 

Exercises/ Phase	**Phase 1**			**Phase 2**			**Phase 3**		
	**Weeks 1-2**			**Weeks 3-5**			**Weeks 5-8**		
**Balance with devices**	Rep	t [s]	RR [s]	Rep	t [s]	RR [s]	Rep	t [s]	RR [s]

Single leg stance on sensory pad	6	30	-						

Lunge with ball rotation	2	15m	-	2	15m	-	2	15m	-

Gluteus bridge on a ball with leg extended	2	30	-						

Ball pull over	15	-	-						

Single leg stance, free leg on swiss ball (knee and hip 90*∗*), gradational weight transfer on free leg	6	15	-						

Jack knife on swiss ball on forearms (knee to the chest)	7	5	-						

Torso twist on ball	15	-	-						

Dumbbell fly on ball (band)	15	-	-						

Assisted ball lunge with squat	10	-	-						

Single leg stance on sensory motor pad (knee 75*∗*)				10R; 10L	-	-	10R; 10L	-	-

Gluteus bridge on a ball with leg extended, pulling the heels toward the buttocks				20	-	-			

Ball pull over, external loading (basketball)				15	-	-			

Single leg stance, free leg on swiss ball (knee and hip ok.90*∗*), equal weight distribution on both legs				4	15	-			

Torso twist on a ball with 3 kg				7R;7L	-	-	2x8	-	-

Dumbbell fly on a ball with 1,5 kg load				15	-	-			

Assisted ball lunge with squat with an extra load				10R; 10L	-	-	2x10	-	-

Gluteus bridge on a ball with leg extended, pulling the heels toward the buttocks, arms cross on the chest							2x 15	-	-

Ball pull over, loading with 3 kg							2x15	-	-

Tall kneeling on swiss ball							3	30	-

Jack knife on swiss ball on hands (ball toward the body, knee extended)				7R;7L	5	-	2x8	5	-

**Rep **= *series x repetitions*; **T **= *time*; **RS **= *rest between series*; **RR **= *rest between repetitions*; **R **= *right limb*; **L **= *left limb*; **R **= *repetitions*; **RE = ***rest before next exercise*; **m**=*metres*.

## Data Availability

The data can be reached through Open ICPSR data repository service under http://doi.org/10.3886/E102440V1
